# Scraps

**Published:** 1889-09

**Authors:** 


### SCRAPS

PICKED UP BY THE ASSISTANT-EDITOR.

---

**The Law of Compensation.**—"It is reported," says the *Evening Wisconsin* "that a Georgia farmer made $100 off an acre planted in watermelons; and a physician in the neighbourhood made $200 off the same acre."—*New York Medical Journal.*

**A Novel Position.**—A subscriber to this *Journal*, looking through its advertisement-pages, and seeing the statement that some manufacturers were prepared to supply "Antipyrin tabloids to the medical profession in bottles," said that he had often heard of the medical profession in their cups, but never in bottles.

**Good all Round.**—"You'll be glad to hear, John," said a farmer to his shepherd one day, "that the University of St. Andrew's has made our minister a doctor." "I am naeways surprised at that," said the shepherd. "Mair than twenty years syne he cured my wife o' a colic. I think he should hae been made a doctor lang syne."

**Vaccination.**—When Jenner began his vaccinations, he, of course, met with much opposition. Caricature and ridicule were freely employed. The following lines, written by a doctor of the time, are very little more unreasonable than many of the arguments used to-day by the opponents of the procedure:

> "O Jenner! thy book, nightly phantasies rousing,
> Full oft makes me quake for my heart's dearest treasure;
> For fancy, in dreams, oft presents them all browsing
> On commons, just like little Nebuchadnezzar.
> *There*, nibbling at thistle, stand Jem, Joe, and Mary.
> On their foreheads, oh, horrible! crumpled horns bud.
> There Tom with his tail, and poor William all hairy,
> Reclined in a corner, are chewing the cud!"

**Is the Taste in the Mouth?**—*The Medical Age* says: "Is it not a little singular that physicians will persist in speaking of the taste in the mouth? A patient was asked the other day if she had a bitter taste in her mouth in the morning. She naïvely replied that when she had a bitter taste it was always in her mouth. That is the only end of the alimentary tract that we know of in which the sense of taste resides." Whereupon *The Medical Record* adds: "If our contemporary will be so particular, we may perhaps venture to remind him that if one comes down to a minute analysis, the taste is not in the mouth in reality at all, but is in the gray matter of the uncinate gyrus. The idea that the taste is in the mouth is simply due to the eccentric projection of the secretion according to the known laws of physio-psychology. We trust that he will make proper explanations to the lady."

**Ideal for a Medical Society.**—In his interesting Presidential address before the Medical Association of Central New York, on May 21st, Dr. William C. Bailey, of Albion, N. Y., presented the following picture of what a Medical Society should be: "A medical society, to serve its highest purpose, ought to be the standard-bearer of the profession; the magnet that will attract the physician from those secluded caverns of habit in which he, above all others, easily obscures himself—a lever that will lift the wheels of his professional chariot out of the ruts of established routine. It should be the physician's Mecca, whither he may journey, a pilgrim consecrating himself anew in his faith. It should be his Olympia, where he may seek social intercourse and friendship, a recreation for his wearied mind and body. It should be to him an Academia, that will broaden his views of life, establish or strengthen religious principles, stimulate and elevate his moral nature. It should be his Athens, whither he may go for increased wisdom, learning in discussion, as did the philosophers of old, obtaining here a knowledge of all progress in operation, treatment, and literature. Finally, it may be his Delphi, the inspiration of his imagination; for in medicine, as in all science, it is often the prophetic finger of Fancy that points the way to the undiscovered. The world is made up of individuals. In the individual we find a unification of various qualities; and his success is assured who correlates—or, if I am permitted, educates—those qualities into such perfect harmony that they attain the highest good. As each component part has its duties, the proper performance of which is essential if the highest degree of perfection possible be desired, so in organised society the individual cannot wholly escape certain obligations that rest upon him, even though, like Byron's Manfred, he exile himself to the solitude of the Alps."—*The Medical and Surgical Reporter.*

**The Black Death.**—The whole period during which the Black Plague raged with destructive violence in Europe was, with the exception of Russia, from the year 1347 to 1350. Most of the great cities suffered incredible losses; above all, Yarmouth, in which 7,052 died, Bristol, Oxford, Norwich, Leicester, York, and London, where, in one burial-ground alone, there were interred upwards of 50,000 corpses, arranged in layers in large pits. Morals were deteriorated everywhere, and the service of God was, in a great measure, laid aside; for, in many places, the churches were deserted, being bereft of their priests. The instruction of the people was impeded; covetousness became general; and when tranquillity was restored, the great increase of lawyers was astonishing, to whom the endless disputes regarding inheritances offered a rich harvest. The sittings of Parliament, of the King's Bench, and of most of the other courts, were suspended as long as the malady raged. Ireland was much less heavily visited than England. The disease seems to have scarcely reached the mountainous districts of that kingdom; and Scotland, too, would perhaps have remained free, had not the Scots availed themselves of the discomfiture of the English, to make an irruption into their territory, which terminated in the destruction of their army, by the plague and by the sword, and the extension of the pestilence, through those who escaped, over the whole country. In Russia the Black Plague did not break out until 1351, after it had already passed through the south and north of Europe. In this country also the mortality was extraordinarily great; and the same scenes of affliction and despair were exhibited, as had occurred in those nations which had already passed the ordeal. The same mode of burial—the same horrible certainty of death—the same torpor and depression of spirits. The wealthy abandoned their treasures, and gave their villages and estates to the churches and monasteries; this being, according to the notions of the age, the surest way of securing the favour of heaven and the forgiveness of past sins. In Russia, too, the voice of nature was silenced by fear and horror. In the hour of danger, fathers and mothers deserted their children, and children their parents. Of all the estimates of the number of lives lost in Europe, the most probable is, that altogether a fourth part of the inhabitants were carried off. It may be assumed without exaggeration that Europe lost during the Black Death 25,000,000 of inhabitants.—Hecker's *Epidemics of the Middle Ages.*

